# Laboratory evaluation of the COBAS MIRA S random access analyser

**DOI:** 10.1155/S1463924691000184

**Published:** 1991

**Authors:** Taweesook Kanluan, Srisanit Intaramanee, Surapon Tangvarasittichai, Orathai Tangvarasittichai, Lerson Suwanton, Sudarat Manochiopinij, Soontharee Tantrarongroj, Pimpan Leangphibul

**Affiliations:** Department of Clinical Chemistry Faculty of Medical Technology Mahidol University Bangkok 10700 Thailand

## Abstract

The random access analyser COBAS MIRA S (Roche Diagnostics) was evaluated for two months. The instrument is a computer-controlled discrete analyser which can be run in a combination profile and single test mode. This instrument has special features, including an automatic cuvette segment changer, a reagent rack cooling system, an external keyboard and monitor, as well as a bar-code facility for the entry of test parameters, worklists and sample identification numbers. Study of within-run and between-run precision gave values of % CV 0.54-3.37 and 0.61-3.65, respectively, for a variety of assays. Linearity testing to the upper limit of each test was also studied and were found to cover the necessary pathological range. Within the two-month period, no major problems were encountered. The instrument required minimum operator attention during operation. Correlation studies with the Hitachi 705 using six clinical chemistry tests (glucose, cholesterol, triglyceride, ALP, AST, ALT) gave correlation coefficients ranging from 0.95-0.99 and slopes of 0.91-1.17.


Journal of Automatic Chemistry, Vol. 13, No. 3 (May-June 1991), pp. 97-100

### Laboratory evaluation of the COBAS MIRA S random access analyser

**Taweesook Kanluan, Srisanit Intaramanee, Surapon Tangvorasittichai, Orathai Tangvorasittichai, Lerson Suwanton, Sudarat Manochiopinij, Soontharee Tantrarongroj and Pimpan Leangphibul**

*Department of Clinical Chemistry, Faculty of Medical Technology, Mahidol University, Bangkok 10700, Thailand*

*The random access analyser COBAS MIRA S (Roche Diagnostics) was evaluated for two months. The instrument is a computer-controlled discrete analyser which can be run in a combination profile and/or single test mode. This instrument has special features, including an automatic cuvette segment changer, a reagent rack cooling system, an external keyboard and monitor, as well as a bar-code facility for the entry of test parameters, worklists and sample identification numbers. Study of within-run and between-run precision gave values of % CV = 0·54–3·37 and 0·61–3·65, respectively, for a variety of assays. Linearity testing to the upper limit of each test was also studied and were found to cover the necessary pathological range. Within the two-month period, no major problems were encountered. The instrument required minimum operator attention during operation. Correlation studies with the Hitachi 705 using six clinical chemistry tests (glucose, cholesterol, triglyceride, ALP, AST, ALT) gave correlation coefficients ranging from 0·95–0·99 and slopes of 0·91–1.17.*

### Introduction

In recent years, random access analysers have attracted considerable interest among clinical chemists in small- to medium-sized laboratories because of their obvious advantages with regard to flexibility, immediate readiness and reliability [1,2]. The random access analyser COBAS MIRA S is an extended version of the well-known COBAS MIRA [3]. This (MIRA S) analyser has an automatic cuvette segment changer, a cooled reagent compartment and improved system software. The filter-photometer system can be monitored in parallel at five different wavelengths: 340, 405, 500, 550 and 600 nm. The working temperature 30 °C is sustained by an air bath and monitored by a thermistor located within the chamber. The software permits the determination of routine clinical chemical analytes by using theoretical calculation factors, calibrators or standard dilution curves. Predilutions of standards and/or samples can be programmed easily. The test results of the determinations are automatically transferred to the 'patient report'. Each patient's data can be recalled and printed whenever needed.

In this paper, the results of an assessment on the analytical performance of COBAS MIRA S are reported. Six routine chemistry tests were selected (table 1) for use in the study of its suitability and usefulness in small- to medium-sized laboratories where routine service and stat tests in both batch and random access mode are performed. The evaluation studies performed included within-run and between-run precision at four analyte concentrations, method linearity and relative accuracy (comparison studies).

*Table 1. Methods and volumes used on the COBAS MIRA S.*

| Test | Method basis | Sample vol. (μl) | Total reagent vol. (μl) |
|---|---|---|---|
| Glucose | UNI-KIT: Hexokinase (UV endpoint) | 4·0 | 200 |
| Cholesterol | UNI-KIT: Cholesterol oxidase (PAP) | 4·0 | 350 |
| Triglyceride | UNI-KIT: Colorimetric end-point (PAP) | 4·0 | 300 |
| ALP | UNI-KIT: Kinetic at 405 nm (IFCC, 37 °C) | 6·0 | 250 |
| AST | UNI-KIT: Kinetic at 340 nm (IFCC, 37 °C) | 16·0 | 145 |
| ALT | UNI-KIT: Kinetic at 340 nm (IFCC, 37 °C) | 16·0 | 145 |

### Materials and methods

### *Instruments*

The COBAS MIRA S was on loan from Roche Diagnostics, Switzerland. All necessary reagents (UNI-KIT) and calibrators were also supplied by Roche. The methods employed for the six analytes, the amount of sample volumes and total reagent volumes are shown in

*Table 2. Methods and volumes for comparison used on the Hitachi 705.*

| Test | Method basis | Sample vol. (μl) | Total reagent* vol. (μl) |
|---|---|---|---|
| Glucose | Glucose oxidase (PAP) | 5·0 | 500 |
| Cholesterol | Cholesterol oxidase (PAP) | 4·0 | 350 |
| Triglyceride | Colorimetric end-point (PAP) | 4·0 | 350 |
| ALP | Kinetic at 405 nm (IFCC, 37 °C) | 5·0 | 350 |
| AST | Kinetic at 340 nm (IFCC, 37 °C) | 20·0 | 385 |
| ALT | Kinetic at 340 nm (IFCC, 37 °C) | 20·0 | 385 |

* All reagents were supplied by Roche Diagnostics.

0142-0453/91 $3.00 © 1991 Taylor & Francis Ltd.

*Table 3. Within-run precision at four analyte concentrations. Coefficients of variation (CV) are given in % (N = 20).*

| | Glucose (mmol/l) | | Cholesterol (mmol/l) | | Triglyceride (mmol/l) | | ALP (I.U./l) | | AST (I.U./l) | | ALT (I.U./l) | |
|---|---|---|---|---|---|---|---|---|---|---|---|---|
| Sample | Mean | CV | Mean | CV | Mean | CV | Mean | CV | Mean | CV | Mean | CV |
| Control N* | 5·58 | 1·47 | 1·96 | 1·68 | 0·29 | 1·81 | 63·08 | 1·48 | 150·10 | 0·63 | 39·11 | 0·97 |
| Control P | 11·96 | 1·58 | 1·81 | 1·04 | 0·37 | 2·11 | 191·80 | 0·89 | 189·30 | 0·54 | 91·53 | 0·72 |
| Ciba-Corning | 4·31 | 1·30 | 3·34 | 0·75 | 0·96 | 1·53 | 72·74 | 1·65 | 26·47 | 2·81 | 23·19 | 2·36 |
| Lyophil | 4·66 | 0·70 | 3·73 | 0·99 | 1·34 | 1·20 | 67·43 | 0·76 | 45·13 | 0·81 | 21·67 | 3·67 |

* Lot K 1736.

*Table 4. Between-run precision at four analyte concentrations. Coefficients of variation (CV) are given in % (N = 20).*

| | Glucose (mmol/l) | | Cholesterol (mmol/l) | | Triglyceride (mmol/l) | | ALP (I.U./l) | | AST (I.U./l) | | ALT (I.U./l) | |
|---|---|---|---|---|---|---|---|---|---|---|---|---|
| Sample | Mean | CV | Mean | CV | Mean | CV | Mean | CV | Mean | CV | Mean | CV |
| Control N* | 5·08 | 1·00 | 1·70 | 1·33 | 0·27 | 3·17 | 64·85 | 3·65 | 134·55 | 0·61 | 33·00 | 0·98 |
| Control P | 12·68 | 1·44 | 1·81 | 1·93 | 0·39 | 3·15 | 211·55 | 1·48 | 194·30 | 1·11 | 90·85 | 1·02 |
| Ciba-Corning-N | 4·80 | 1·33 | 3·49 | 1·74 | 0·94 | 1·86 | 84·25 | 3·51 | 27·20 | 2·82 | 24·90 | 2·81 |
| Lyophil | 5·08 | 0·62 | 3·89 | 1·22 | 1·36 | 1·21 | 78·10 | 2·78 | 46·25 | 1·38 | 24·90 | 3·17 |

* Lot K 1440.

*Table 5. Linearity of assays as performed by the COBAS MIRA S.*

| Test | Determined range of linearity |
|---|---|
| Glucose | 0·56–26·42 mmol/l |
| Cholesterol | 0–18·10 mmol/l |
| Triglyceride | 0–6·58 mmol/l |
| ALP | 0–975 I.U./l |
| AST | 0–397 I.U./l |
| ALT | 0–428 I.U./l |

*Table 6. Linear regression statistics for the COBAS MIRA S (y-axis) against the Hitachi 705 (x-axis).*

| Test | Slope | y-intercept | Correlation coefficient | N |
|---|---|---|---|---|
| Glucose | 0·91 | 14·18 | 0·98 | 126 |
| Cholesterol | 0·98 | −11·76 | 0·99 | 127 |
| Triglyceride | 1·00 | −0·79 | 0·99 | 147 |
| ALP | 1·17 | −2·36 | 0·99 | 120 |
| AST | 1·04 | 1·44 | 0·99 | 121 |
| ALT | 1·01 | 0·27 | 0·99 | 124 |

table 1. All reagents were prepared according to the manufacturer's instructions. The analyser used in the comparison study was the Hitachi 705 (Boehringer Mannheim, Germany), this was the instrument used by the laboratory before the arrival of the COBAS MIRA S. All methods used on the Hitachi 705 were performed and calibrated with Roche Diagnostics reagents, calibrators and controls. Methods, sample volumes and total reagent volumes are shown in table 2.

### *Specimens*

The following commercially available control sera were used in the precision studies according to the manufacturer's instructions: (1) control serum N: lot K 1736 and lot K 1440 and control serum P: lot C 2741 Roche Diagnostics; (2) Ciba-Corning: lot Ch-B: 036701, USA and (3) Lyophil: lot 0186005 (locally produced, distributed by the faculty of Medical Technology, Mahidol University).

More than 120 patient samples (low, normal and above-normal levels) were selected for comparison studies. Half of each specimen was used for assays in the COBAS MIRA S, while the other half was used for assays in the Hitachi 705. The above-normal concentration specimens were used in the linearity studies.

### *Calibration*

The COBAS MIRA S and the Hitachi 705 were calibrated with Roche Diagnostics calibration material.

### Results

### *Precision*

The evaluation of the performance of COBAS MIRA S took two months. Close supervision and appropriate training were given initially by Roche Diagnostics (Thailand) Ltd. Within-run and between-run precisions were assessed. Six clinical chemical analytes were selected; glucose, cholesterol, triglyceride, alkaline phosphatese (ALP), aspartate aminotransferase (AST) and alanine aminotransferase (ALT). For within-run studies, four commercially available control sera were analysed 20 times each in single batches, while the between-run

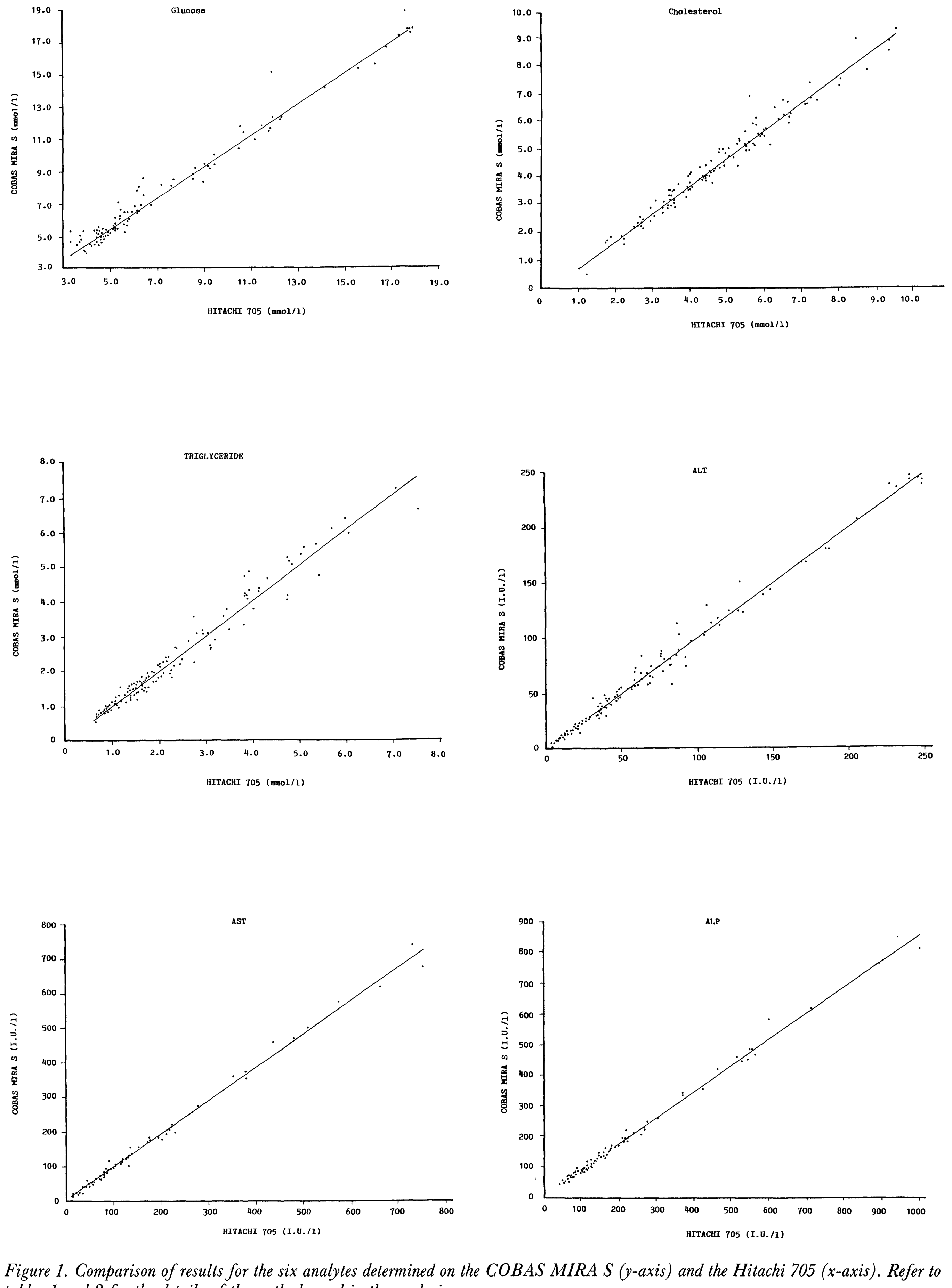

*Figure 1. Comparison of results for the six analytes determined on the COBAS MIRA S (y-axis) and the Hitachi 705 (x-axis). Refer to tables 1 and 2 for the details of the methods used in the analysis.*

precision was estimated by running the same sera on 20 separate days. The values of the imprecision within-run and between-run obtained are shown in tables 3 and 4, respectively. The overall % CV of both precision studies gave values of not greater than 3%.

*Linearity*

High value samples that greatly exceed the upper limit of determinations were appropriately diluted with isotonic saline for use in linearity studies of each method. The upper limit of each analyte obtained from the study is shown in table 5. The upper limits were in close agreement with the expected range for each analyte as claimed by the manufacturer.

*Accuracy*

More than 120 serum samples, representing both normal and abnormal levels were divided and run simultaneously in the COBAS MIRA S and the Hitachi 705. The comparison data which comprise slopes, intercepts and correlation coefficients are shown in table 6 and figure 1. The Hitachi 705 is the independent variable and COBAS MIRA S is the dependent variable. Good agreement was found in all cases.

### Discussion

The purpose of this study was to assess the overall performance of the COBAS MIRA S in a clinical laboratory performing routine tests.

The precision data in table 3 and 4 show that the range of precision determined from COBAS MIRA S is within the acceptable limits. The good precision with % CV, in the range of 0·5–3·6, may be due to the pipetting system, where the reagent and sample probe are washed, and to the individual reaction cuvette used in the test measurement.

The comparison of the test results obtained on serum samples with COBAS MIRA S and with the Hitachi 705 shows that the degree of correlation was very good ($r$ ranging from 0·95–0·99).

In laboratories where ambient controlled temperature is often in the range of 25–30 °C, the rack cooling device is a very useful compartment to hold temperature-sensitive reagents, especially where large batch of reagents are needed to facilitate the 'true walk-away' facility (provided by the manufacturers for 312 analyses to be determined in one run and facilitated by the automatic cuvette segment changer).

The instrument performed well over the evaluation period. No major problems with regards to instrument failure occurred during the study. The operator's manual and guidelines for trouble-shooting were easy to follow.

In conclusion, the COBAS MIRA S is a flexible, convenient and easy-to-use analyser for either batch or random access work. Its design and operational simplicity provides reliable analytical data, as demonstrated by good correlation with the existing instrument. Unique, cooled reagent reservoirs and sealed serum cups make the COBAS MIRA S an ideal instrument for use in small to medium-sized laboratories where efficient output of laboratory data is required.

### Acknowledgement

The authors wish to express their sincere thanks to Roche Diagnostics (Thailand) for the reagents and technical support.

### References

1. Bonini, P., Ceriotti, F. and Franzini, C., *Journal of Automatic Chemistry,* **10** (1988), 167.
2. Beny, D. J. and Price, C. P., *Journal of Automatic Chemistry,* **11** (1989), 15.
3. Lifshity, M. and De Cresce, R., *Laboratory Medicine,* **17** (1986), 291.

